# Healthcare-associated infections in Dutch hospitals during the COVID-19 pandemic

**DOI:** 10.1186/s13756-022-01201-z

**Published:** 2023-01-05

**Authors:** Janneke D. M. Verberk, Tjallie I. I. van der Kooi, Nynke A. Kampstra, Naomi Reimes, Stephanie M. van Rooden, Titia E. M. Hopmans, Suzanne E. Geerlings, Sabine C. de Greeff

**Affiliations:** 1 Department of Epidemiology and Surveillance, Centre for Infectious Diseases Control, National Institute for Public Health and the Environment, Antonie Van Leeuwenhoeklaan 9, 3721 MA Bilthoven, The Netherlands; 2 Department of Medical Microbiology and Infection Prevention, University Medical Centre Utrecht, Utrecht, The Netherlands; 3 Department of Internal Medicine, Amsterdam University Medical Centres, Infection and Immunity, Amsterdam Public Health, University of Amsterdam, Amsterdam, The Netherlands

**Keywords:** Healthcare-associated infections, Covid-19, Pandemic, Surveillance

## Abstract

**Background:**

During the COVID-19 pandemic hospitals reorganized their resources and delivery of care, which may have affected the number of healthcare-associated infections (HAIs). We aimed to quantify changes in trends in the number of HAIs in Dutch hospitals during the COVID-19 pandemic.

**Methods:**

National surveillance data from 2016 to 2020 on the prevalence of HAIs measured by point prevalence surveys, and the incidence of surgical site infections (SSIs) and catheter-related bloodstream infections (CRBSIs) were used to compare rates between the pre-pandemic (2016–February 2020) and pandemic (March 2020–December 2020) period.

**Results:**

The total HAI prevalence among hospitalised patients was higher during the pandemic period (7.4%) compared to pre-pandemic period (6.4%), mainly because of an increase in ventilator-associated pneumonia (VAP), gastro-intestinal infections (GIs) and central nervous system (CNS) infections. No differences in SSI rates were observed during the pandemic, except for a decrease after colorectal surgeries (6.3% (95%-CI 6.0–6.6%) pre-pandemic versus 4.4% (95%-CI 3.9–5.0%) pandemic). The observed CRBSI incidence in the pandemic period (4.0/1,000 CVC days (95%-CI 3.2–4.9)) was significantly higher than predicted based on pre-pandemic trends (1.4/1000 (95%-CI 1.0–2.1)), and was increased in both COVID-19 patients and non-COVID-19 patients at the intensive care unit (ICU).

**Conclusions:**

Rates of CRBSIs, VAPs, GIs and CNS infections among hospitalised patients increased during the first year of the pandemic. Higher CRBSI rates were observed in both COVID-19 and non-COVID-19 ICU population. The full scope and influencing factors of the pandemic on HAIs needs to be studied in further detail.

**Supplementary Information:**

The online version contains supplementary material available at 10.1186/s13756-022-01201-z.

## Background

When the World Health Organization on March 11, 2020 officially declared the coronavirus disease 2019 (COVID-19) a global pandemic [1], COVID-19 hospitalisations in the Netherlands were already increasing rapidly. The high influx of patients impacted the critical care capacity, work processes, and availability and use of protective equipment in hospitals [2–5]. To handle the pressure and high demand of care during this crisis, hospitals reorganised their resources and delivery of care [6]. For example, elective surgeries were postponed or cancelled, intensive care unit (ICU) bed capacity was scaled up, the ratio of healthcare workers allocated to patients was reduced, external staff was hired, and changes to daily care routines, such as the frequency of patient washing, was reduced [7–9].

During this pandemic situation, attention to infection prevention and control (IPC) measures may have been deprived given the high work pressure, or redirected towards the prevention of SARS-CoV-2 transmission [10]. In addition, patients hospitalised with COVID-19 are known for having comorbidities, long hospital stays and complex care with multiple invasive devices, putting them at higher risk for healthcare-associated infections (HAIs) [11]. Hence, an increase of HAIs could be expected and is also reported by previous studies [12, 13]. On the other hand, hospitals applied strict, aggressive IPC measures to prevent within-hospital transmission of SARS-CoV-2. As a result, a positive (indirect) effect on HAI occurrence can be expected as well and has been reported by others [14–16].

Given these contrasting findings, there is need for adequate HAI reporting not limited to COVID-19 cohorts only, with sufficient historical data to allow pre-pandemic comparisons. The aim of this study was to quantify trends in the number of HAIs in Dutch hospitals during the COVID-19 pandemic, using national surveillance that continued during the pandemic. Second, HAI types were compared between COVID-19 patients versus non-COVID-19 patients.

## Methods

### Study design and data sources

In this retrospective cohort study, data were derived from the Dutch national nosocomial surveillance network (PREZIES). In short, acute care hospitals voluntarily participate in one or more of the three surveillance modules targeting different HAIs: (1) bi-annually Point Prevalence Surveys (PPS) performed in March and October in which the prevalence of all type of HAIs are measured in all admitted patients (excluding patients admitted to psychiatry and day-care units), (2) Surgical site infection (SSI) incidence surveillance on targeted procedures (see Additional file 1: Table S1 for an overview of the procedures), and (3) hospital-wide catheter-related bloodstream infection (CRBSI) incidence surveillance in patients with a central venous catheter (CVC) in place for ≥ 48 h. For each module, infection control practitioners in each hospital manually review medical records retrospectively according to the national surveillance protocols and annotate which patients meet infection case definitions. The surveillance protocols and case definitions are based on the (European) Centres for Disease Control and Prevention and are described elsewhere [17–19]. Only in the PPS and CRBSI modules information was collected about whether the patient was admitted to the hospital due to COVID-19 (positive test at admission). Hospitals that reported their surveillance data yearly to PREZIES over the years 2016–2020 were included in this study and used to evaluate the infection rates during the pre-pandemic and pandemic period.

### Definition pre-pandemic and pandemic period

Based on COVID-19 hospitalisation rates in the Netherlands, the PPS surveys of 2016–2019 were defined as pre-pandemic and the surveys of March and October 2020 were defined as the pandemic period. Data from the SSI and CRBSI modules were divided in pre-pandemic (January 2016 to February 2020) and pandemic (from 1st of March 2020 to December 2020).

### Statistical analyses

Per module, patient-, surgery-, or CVC- related characteristics were reported and compared between the pre-pandemic and pandemic period, using a chi-square test for categorical variables and Mann–Whitney U test for continuous variables. Thereafter, we quantified the number of HAIs during the pandemic. For PPS data, the difference in observed HAI rates between the pre-pandemic and pandemic period was tested using chi-square 2-tailed test with Yates’ correction.

For the SSI and CRBSI incidence, we estimated the expected infection rates for the pandemic period based on pre-pandemic data and compared this with the actual observed rates in the pandemic period. To estimate the expected incidence rate for SSI, the National Nosocomial Infections Surveillance System (NNIS) risk index in pre-pandemic data was used to predict the risk of SSI for each NNIS category for the pandemic period (Additional file 1: Figure S1). The NNIS risk index, ranging from 0 to 3, is composed of 1 point for each of the following criteria: wound class classified as contaminated or infected; American Society of Anaesthesiologists (ASA) score of 3, 4, or 5; and an operation duration above the 75th percentile [20]. The predicted infection rate was compared with the observed infection rate using a chi-square test. In addition, two sensitivity analyses for SSI were performed. First, the same analyses were repeated for deep SSI only, with the rationale that superficial SSIs may have been missed during follow-up in the pandemic period: patients avoided contact with healthcare providers afraid of becoming infected with SARS-CoV-2, patients did not want to be a burden on the system, and follow-up appointments were replaced by remote care because of stay-at-home orders [9]. Second, trends in SSI incidence rates were checked per surgical specialty. In case an increasing or decreasing trend was observed pre-pandemic, the expected SSI rate was recalculated based on 2019 data only. To estimate the expected CRBSI incidence per 1000 CVC days in the pandemic period, the mean pre-pandemic incidence per 1000 CVC days for each of the three application-based categories (total parenteral nutrition (TPN); dialysis; and the remaining other applications) was multiplied with the pandemic number of CVCs in each category (Additional file 1: Figure S1). The predicted and observed incidence rates were compared using a mid-P exact test. Last, differences in patient characteristics, medical device use, and HAIs were investigated in COVID-19 patients versus non-COVID-19 patients based on PPS and CRBSI data, by using a chi-square test or Mann–Whitney test. A *p* value of < 0.05 was considered statistically significant and analyses were performed using SAS version 9.4 software (SAS Institute, Cary, NC).

## Results

Table 1 shows the number of hospitals participating in the three different modules, per year. The number of hospitals reporting PPS data during the pandemic year 2020 was less than half compared with previous years. Subsequent analyses were performed for the PPS, SSI, and CRBSI module, using data from 10, 51, and 11 hospitals respectively that reported their yearly surveillance data in 2016–2020 to PREZIES (Table 1). In these hospitals, the absolute annual number of admissions (PPS module) and surgeries (SSI module) was lower in 2020 compared to previous years, while there was a slight increase in the number of inserted CVCs (CRBSI module).

**Table 1:** Overview of hospitals included in this study

	Number of hospitals reporting data to PREZIES	Number of hospitals included in this study reporting data each year in 2016–2020 (general/teaching/academic)	Number of patients, surgeries, and CVCs included, respectively
*PPS module*		10 (6/1/3)	
2016	40	NA	4036
2017	37	NA	3956
2018	27	NA	3841
2019	30	NA	4273
2020	11	NA	3124
*SSI module*		51 (33/16/2)	
2016	84	NA	48,760
2017	81	NA	50,487
2018	75	NA	51,816
2019	68	NA	56,286
2020	66	NA	45,656
*CRBSI module*		11 (8/3/0)	
2016	31	NA	2454
2017	28	NA	2030
2018	26	NA	1735
2019	21	NA	2019
2020	18	NA	2286

*PPS* point prevalence survey, *n* number, *SSI* surgical site infection, *NA* not applicable, *CRBSI* catheter-related bloodstream infection, *CVCs* central venous catheters

### Healthcare-associated infections during the first pandemic year

#### Point prevalence survey results

During the pandemic period, a higher proportion of hospitalised patients was male, patients had slightly higher McCabe scores and more ICU admissions were observed (Table 2). The proportion of patients having a medical device increased during the pandemic period, in particular the use of CVCs. The proportion of patients with antibiotic treatment at the time of the survey was slightly higher during the pandemic (42.6%) versus pre-pandemic (37.7%; *p* < 0.01). The total HAI prevalence was higher during the pandemic period compared to pre-pandemic period, mainly because of an increase in gastro-intestinal infections and infections of the central nervous system (Tables 3, 4). The proportion of patients with lower respiratory tract infections (LRTIs) in the pandemic period was similar compared to pre-pandemic, however, a larger proportion was associated with mechanical ventilation (ventilator-associated pneumonia (VAP), 22.5% pandemic versus 13.5% pre-pandemic, Table 4).

**Table 2:** Patient-, surgery-, and central venous catheter characteristics

	Pre-pandemic	Pandemic	*p* value
*PPS module*	16,106 patients	3124 patients	
Age in years [median, (IQR)]	64.8 (32.9)	63.9 (34.2)	< 0.01
Age group (n (%))			< 0.01
< 1 year	1191 (7.3)	272 (8.7)	
1–19 year	804 (5.0)	163 (5.2)	
20–29 year	708 (4.4)	126 (4.0)	
30–39 year	1032 (6.4)	194 (6.2)	
40–49 year	1080 (6.7)	211 (6.8)	
50–59 year	1951 (12.1)	405 (13.0)	
60–69 year	2972 (18.5)	556 (18.1)	
70–79 year	3430 (21.3)	705 (22.6)	
80–89 year	2412 (15.0)	406 (13.0)	
≥ 90 year	526 (3.3)	76 (2.4)	
Sex [male (n (%))]	8060 (50.0)	1625 (52.0)	0.04
Specialty [n (%)]			< 0.01
Cardiology	1654 (10.3)	304 (9.7)	
Surgery	2284 (14.2)	434 (13.9)	
Internal medicine	1908 (11.8)	332 (10.6)	
Paediatrics	1140 (7.1)	216 (6.9)	
Respiratory medicine	1285 (8.0)	235 (7.6)	
Other	7835 (48.6)	1603 (51.3)	
McCabe [n (%)]			< 0.01
Non-fatal (> 5 year)	11,615 (72.1)	2141 (68.5)	
Ultimately fatal (1–5 year)	1394 (8.7)	311 (10.0)	
Rapidly fatal (< 1 year)	308 (1.9)	69 (2.2)	
Unknown	2789 (17.3)	603 (19.3)	
ICU [n (%)]			< 0.01
Yes	1170 (7.3)	281 (9.0)	
No	14,936 (92.7)	2843 (91.0)	
Medical devices [n (%)]^a^			
Urethral catheter	3374 (20.9)	711 (22.8)	0.02
Peripheral catheter	9011 (56.0)	1767 (56.6)	0.5
Mechanical ventilation	482 (3.0)	128 (4.2)	< 0.01
Central venous catheter	1,572 (9.8)	458 (14.7)	< 0.01
Antibiotics [n (%)]			< 0.01
Yes	6065 (37.7)	1330 (42.6)	
No	10,041 (62.3)	1794 (57.4)	
*SSI module*	217,212 surgeries	35,793 surgeries	
Age in years [median (IQR)]	67.7 (57.5–74.7)	67.3 (56.4–74.5)	< 0.01
Sex [male (n (%))]	67.137 (31.6)	11.193 (34.0)	< 0.01
Body mass index [median (IQR)]	27.3 (24.4–30.8)	27.2 (24.3–30.7)	< 0.01
Length of stay in days (median (IQR))	2 (0–274)	1 (0–95)	< 0.01
Duration of surgery in minutes [median (IQR)]	62 (47–80)	59 (44–76)	< 0.01
ASA classification [n (%)]			< 0.01
1	38,062 (17.5)	5083 (14.2)	
2	130,422 (60.0)	21,954 (61.3)	
3	38,025 (17.5)	7223 (20.2)	
4	1138 (0.5)	217 (0.6)	
5	58 (0.0)	4 (0.0)	
Unknown/NA	9507 (4.4)	1312 (3.6)	
NNIS index [n (%)]			< 0.01
0	139,092 (64.0)	21,199 (59.2)	
1	59,217 (27.3)	11,009 (30.8)	
2	8891 (4.1)	2186 (6.1)	
3	248 (0.1)	66 (0.2)	
Unknown/NA	9764 (4.5)	1333 (3.7)	
Type of surgery [n (%)]			< 0.01
Cardiothoracic surgery	5596 (2.6)	948 (2.6)	
Mamma surgery	24,556 (11.3)	4080 (11.4)	
Colon surgery	26,832 (12.4)	4770 (13.3)	
Orthopaedic surgery	140,821 (64.8)	22,353 (62.5)	
Obstetrics	15,465 (7.1)	2896 (8.1)	
Neurosurgery	3942 (1.8)	746 (2.1)	
*CRBSI module*	8595 patients (10,546 CVCs)	1929 patients (2614 CVCs)	
Age in years [median (IQR)]	69.5 (60.3–76.5)	68.6 (59.1–74.5)	< 0.01
Sex [male (n (%))]	5044 (58.7)	1259 (65.3)	< 0.01
Number of CVCs per patient [median (IQR)]	1.2 (1–1)	1.3 (1–1)	< 0.01
CVC days [median (IQR)]	5 (3–8)	6 (4–9)	< 0.01
ICU [n (%)]			< 0.01
Yes	6574 (76.5)	1591 (82.5)	
No	2021 (23.5)	338 (17.5)	
CVC use [n (%)]^b^			
Total parenteral nutrition	1,889 (17.9)	428 (16.4)	0.06
Antibiotics	5037 (47.8)	1624 (62.1)	< 0.01
Dialysis	1191 (11.3)	312 (11.9)	0.36
Hemodynamic monitoring	5466 (51.8)	1500 (57.4)	< 0.01
Other	1861 (17.6)	304 (11.6)	< 0.01

*PPS* point prevalence survey, *n* number, *ICU* intensive care unit, *SSI* surgical site infection, *IQR* interquartile range, *NA* not applicable, *NNIS* National Nosocomial Infections Surveillance System, *CRBSI* catheter-related bloodstream infection, *CVC* central venous catheter

^a^Patients can have multiple devices at the same time. Percentages are calculated as the proportion of patients with a specific device out of the total number of patients

^b^Patients can have a CVC for multiple applications. Percentages are calculated as the proportion of CVCs for a specific use out of all CVCs

**Table 3:** Infection rates pre-pandemic, predicted infection rates during pandemic, and observed infection rates during the pandemic

	Pre-pandemic [% (95%-CI)]	Predicted [% (95%-CI)]	Pandemic [% (95%-CI)]
*HAI prevalence (by PPS)*			
Total HAI (by PPS)	6.4 (6.0–6.8)	NA	7.4 (6.5–8.3)*
SSIs	2.2 (1.9–2.4)	NA	2.3 (1.9–2.9)
RTIs	1.2 (1.1–1.4)	NA	1.4 (1.1–1.9)
BSIs (primary and secondary)	1.3 (1.1–1.5)	NA	1.4 (1.1–1.9)
UTIs	0.8 (0.7–1.0)	NA	0.8 (0.5–1.2)
Other	0.9 (0.8–1.1)	NA	1.4 (1.1–1.9)*
*SSI incidence*			
Total	2.1 (2.0–2.1)	2.1 (2.0–2.3)	1.8 (1.6–1.9)*
Cardiothoracic surgery	1.7 (1.4–2.1)	1.7 (1.4–2.1)	1.9 (1.2–3.0)
Mamma surgery	3.8 (3.6–4.0)	4.0 (3.4–4.6)	3.4 (2.9–4.0)
Colon surgery	6.3 (6.0–6.6)	6.5 (5.9–7.3)	4.4 (3.9–5.0)*
Orthopaedic surgery	1.1 (1.0–1.1)	1.2 (1.0–1.3)	1.0 (0.9–1.1)
Obstetrics	1.4 (1.2–1.6)	1.4 (1.0–1.9)	1.3 (1.0–1.8)
Neurosurgery	1.0 (0.7–1.4)	0.7 (0.4–1.3)	0.8 (0.4–1.7)
*CRBSI incidence*			
	1.6 (1.3–2.0)	1.4 (1.0–2.1)	4.0 (3.2–5.0)*

*95%-CI* 95% confidence interval, *HAI* healthcare-associated infection, *PPS* point prevalence survey, *SSI* surgical site infection, *CRBSI* catheter-related bloodstream infection, *RTIs* respiratory tract infections, *BSIs* bloodstream infections, *UTIs* urinary tract infections, *NA* not applicable

*Statistically significant different from predicted rates

**Table 4:** Distribution of healthcare-associated infections in pre-pandemic and pandemic PPS cohort

	Pre-pandemic n = 16,106 [n (%)]	Pandemic n = 3124 [n (%)]
Total HAI (by PPS)	n = 1028 (6.4%)*	n = 230 (7.4%)*
SSIs	347 (33.8)	73 (31.7)
RTIs	202 (19.7)	45 (19.6)
Of which lower RTIs	177 (87.6)	40 (88.9)
Associated with mechanical ventilation (VAP)	24 (13.5)*	9 (22.5)*
BSIs	205 (20.0)	45 (19.6)
Of which catheter-related	44 (4.3)	6 (2.6)
UTIs	134 (13.0)	25 (10.9)
Of which catheter-related	81 (7.9)	19 (8.2)
GTIs	37 (3.6)*	16 (7.0)*
Skin infections	35 (3.4)	7 (3.0)
Mouth infections	16 (1.6)	5 (2.2)
Central nervous system infections	13 (1.3)*	7 (3.0)*
Cardiovascular infections	12 (1.2)	3 (1.3)
Bone infections	11 (1.1)	0 (0.0)
Other systemic infections	8 (0.8)	0 (0.0)
Reproductive tract infections	5 (0.5)	2 (0.9)
Eye infections	2 (0.2)	2 (0.9)
Ear infections	1 (0.1)	0 (0.0)

*PPS* point prevalence survey, *HAIs* healthcare-associated infections, *SSIs* surgical site infections, *RTIs* respiratory tract infections, *VAP* ventilator-associated pneumonia, *BSIs* bloodstream infections, *UTIs* urinary tract infections, *GTIs* Gastro-intestinal infections. Percentages are presented as % out of total HAIs

*Statistically significant

#### Surgical site infections

**Fig. 1:**
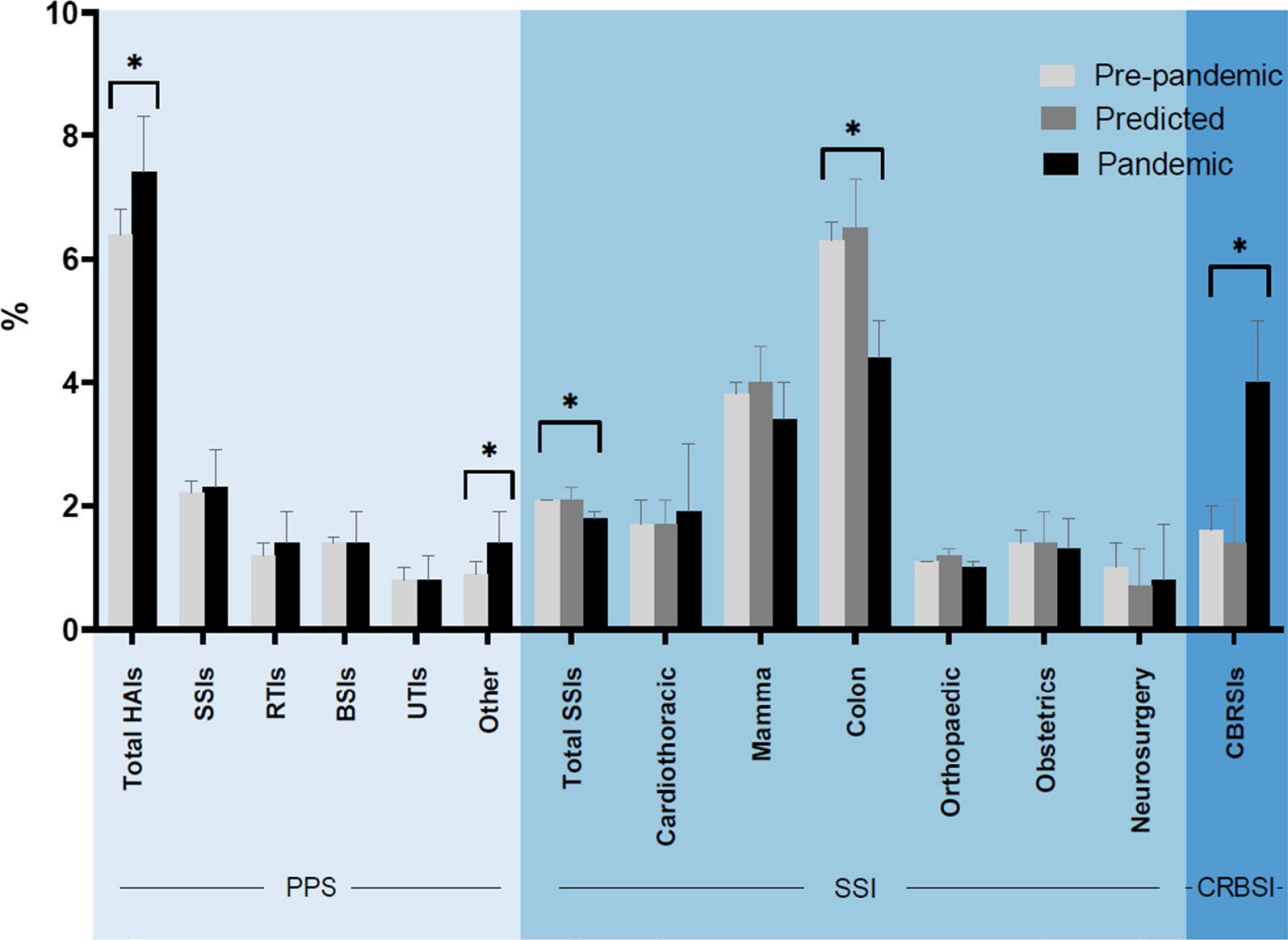
Infection rates pre-pandemic, and predicted and observed infection rates during the pandemic period. *HAIs* healthcare-associated infections, *PPS* point prevalence survey, *SSIs* surgical site infections, *RTIs* respiratory tract infections, *BSIs* bloodstream infections, *UTIs* urinary tract infections, *CRBSIs* catheter-related bloodstream infections

Within the SSI module, 217,212 surgeries were included in the pre-pandemic period versus 35,793 surgeries during the pandemic. Compared to the pre-pandemic period, patients operated during the pandemic period were more often of the male gender, had slightly higher ASA- and NNIS scores and had shorter hospital stays (Table 2). The observed SSI incidence for all type of surgeries combined in the pandemic period was significantly lower than predicted (1.8% versus 2.1%, respectively) (Fig. 1 and Table 3). When stratified by surgery type, only the SSI incidence after colon surgery was significantly lower during the pandemic (*p* < 0.01; Table 3). During 2016–2019, already a decreasing trend in SSI incidence after colorectal surgeries was observed (7.2%; 7.2%; 6.3%; 5.0%, respectively), while the proportion of closed procedures increased (*p* < 0.01, Additional file 1: Figure S2). When calculating the expected SSI incidence after colorectal surgery based on 2019 data only, the SSI rate in de pandemic was as predicted (predicted SSI rate: 5.1%; 95%-CI 4.5–5.8, observed SSI rate: 4.4%; 95%-CI: 3.9–5.0, *p* = 0.1). Sensitivity analysis comparing observed and expected incidence of deep SSI only showed similar results (Additional file 1: Table S2).

#### Catheter-related bloodstream infections

During the pandemic period, patients with a CVC were slightly younger, more often of the male gender and more often admitted to the ICU compared to the pre-pandemic period. During the pandemic period, the number of inserted CVCs per patient was slightly higher and the CVC duration was longer. CVCs were more frequently used for antibiotics and hemodynamic monitoring and less often for TPN (Table 2). The observed CRBSI incidence of 4.0/1000 CVC days (95%-CI 3.2–4.9/1000) in the pandemic period was significantly higher than the predicted rate of 1.4/1000 CVC days (95%-CI 1.0–2.1/1000; *p* < 0.01) (Fig. 1).

### Healthcare-associated infections within COVID-19 patients

Within the PPS module, COVID-19 status was only registered during the survey in October 2020: 50 (6.6%) patients were SARS-CoV-2 positive during admission and were compared with 713 (93.4%) non-COVID-19 patients. COVID-19 patients were more often admitted to the ICU, had more often medical devices and antibiotic use (Table 5). A significantly higher HAI prevalence was observed in this patient group as compared to non-COVID-19 patients (12% versus 0.4% respectively, *p* < 0.01), with bloodstream infections (BSI) as the most predominant manifestation (Additional file 1: Table S3).

**Table 5:** Differences in COVID-19 patients versus non-COVID patients admitted to the hospital, March 2020–December 2020

	COVID-19 patient	Non-COVID-19 patient	*p* value
PPS module [n (%)]	n = 50	n = 713	
Age in years [median, (IQR)]	71.7 (20.7)	66.8 (30.0)	< 0.01
Sex [male (n (%))]	32 (64.0)	355 (49.8)	0.05
Specialty [n (%)]			< 0.01
Cardiology	1 (2.0)	81 (11.4)	
Surgery	1 (2.0)	125 (17.5)	
Internal medicine	8 (16.0)	110 (15.4)	
Paediatrics	0 (0.0)	52 (7.3)	
Respiratory medicine	25 (50.0)	53 (7.4)	
Other	15 (30.0)	292 (41.0)	
McCabe [n (%)]			0.93
Non-fatal (> 5 year)	44 (88.0)	620 (87.0)	
Ultimately fatal (1–5 year)	5 (10.0)	74 (10.4)	
Rapidly fatal (< 1 year)	1 (2.0)	13 (1.8)	
Unknown	0 (0.0)	6 (0.8)	
ICU [n (%)]			< 0.01
Yes	13 (26.0)	31 (4.4)	
No	37 (74.0)	682 (95.6)	
Medical devices [n (%)]^a^			
Urethral catheter	10 (20.0)	147 (20.6)	0.36
Peripheral catheter	39 (78.0)	456 (64.0)	0.04
Mechanical ventilation	5 (10.0)	8 (1.2)	< 0.01
Central venous catheter	5 (10.0)	47 (6.6)	0.35
Antibiotics [n (%)]			< 0.01
Yes	32 (64.0)	266 (37.3)	
No	18 (36.0)	447 (62.7)	
HAIs [% (95%-CI)]	12 (5.6–23.8)	0.4 (0.1–1.2)	< 0.01
CRBSI module [n (%)]	n = 367	n = 708	
Age in years [median (IQR)]	66.2 (57.0–71.8)	69.3 (58.3–75.1)	< 0.01
Sex [male (n (%))]	288 (78.5)	435 (61.4)	< 0.01
Number of CVCs per patient [median (IQR)]	1.8 (1–2)	1.3 (1–1)	< 0.01
CVC days [median (IQR)]	7 (5–10)	6 (4–9)	< 0.01
ICU [n (%)]			< 0.01
Yes	350 (95.4)	518 (73.2)	
No	17 (4.6)	190 (26.8)	
Length of ICU stay in days [median (IQR)]	18 (8–33)	4 (2–11)	< 0.01
CVC use [n (%)]^b^			
Total parenteral nutrition	37 (5.6)	200 (21.9)	< 0.01
Antibiotics	454 (69.3)	523 (57.2)	< 0.01
Dialysis	92 (14.0)	130 (14.2)	0.94
Hemodynamic monitoring	319 (48.7)	441 (48.2)	0.88
Other	98 (15.0)	144 (15.8)	0.72
CRBSI per 1000 CVC days (95%-CI)	8.1 (5.9–10.8)	3.4 (2.2–5.0)	< 0.01

For PPS, COVID-19 status was only measured in the survey of October 2020. For CRBSI, COVID-19 status was reported by 9 out of 11 hospitals for the majority (56.2%) of the patients: 19.2% were COVID-19 patients, 37.0% non-COVID-19 and for the remaining 43.8% within the CRBSI module, COVID-19 status was unknown

^a^Patients can have multiple devices at the same time. Percentages are calculated as the proportion of patients with a specific device out of the total number of patients

^b^Patients can have a CVC for multiple applications. Percentages are calculated as the proportion of CVCs for a specific use out of all CVCs

A total of 9 out of 11 hospitals participating in the CRBSI module reported whether the patient was admitted to the hospital due to COVID-19. These COVID-19 patients were more often male, were slightly younger in age, and had significant longer ICU length of stay compared to non-COVID-19 patients with a CVC during the pandemic period. In addition, COVID-19 patients had more CVCs inserted and with a longer duration (Table 5). The CVC was more often used for antibiotics and less for TPN compared to non-COVID-19 patients. The CRBSI incidence was 8.1/1000 CVC days (95%-CI 5.9–10.8) in COVID-19 patients compared to 3.4/1000 (95%-CI 2.2–5.0) in patients without COVID-19 (*p* < 0.01). When stratifying the COVID-19 patients to ICU and non-ICU, CRBSI rates were 7.8/1000 CVC days (95%-CI 5.6–10.7) and 11.1 (95%-CI 5.0–24.7) respectively. When stratifying the non-COVID-19 patients to ICU and non-ICU, CRBSI rates were 4.8/1000 CVC days (95%-CI 3.0–7.6) and 1.7 (95%-CI 0.7–4.0) respectively. The CRBSI incidence for non-COVID-19 patients in the ICU (4.8/1000) was significantly higher compared to pre-pandemic years (0.7/1000; 95%-CI 0.5–1.1) as well.

## Discussion

During the first pandemic year CRBSIs, VAPs, gastro-intestinal- and central nervous system infections occurred more frequently among hospitalised patients, while SSIs and catheter-associated urinary tract infection (CAUTI) rates remained stable. HAIs occurred more often in COVID-19 patients, however, in non-COVID-19 patients admitted to the ICU an sevenfold increase of CRBSI was observed during the pandemic compared to pre-pandemic as well.

Regarding SSI, less surgeries were performed in 2020 and the patients that have been operated had slightly higher ASA and NNIS scores compared to previous years, possibly explained by prioritising urgent procedures during the pandemic period. Although this patient population may be more likely to develop SSIs, no increase in incidence was observed. Remarkable is the relative high number of laparoscopic colon surgeries during the pandemic, which may be induced by policies to relieve ICU capacity and the shift to minimally invasive surgery to protect operating room personnel from SARS-CoV-2 aerosol transmission [21]. Future data will show whether open surgery had been replaced during the pandemic by closed surgery, or whether the open surgeries were postponed.

The findings of this study are in line with previous research: several studies reported increases during the pandemic in among others CRBSIs, BSIs, and VAPs [12, 13, 22–25]. The PPS data showed that the prevalence of LRTIs did not change, however the proportion of LRTIs associated with ventilation increased, likely due to the increased use of mechanical ventilation [26]. Importantly, the work pressure, burden and influx of COVID-19 patients was not constant throughout 2020: COVID-19 surges varied during the year, by region and by hospital [27]. Especially for the PPS, the timing of the surveys (March and October) may not have paralleled the COVID-19 surges and circumstances and therefore may have underestimated potential effects: we did not find any increase in CRBSIs or CAUTIs in the PPS data while this was reported by others [23, 24]. Within the CRBSI module, the number of CRBSI events was too low to perform sub-analyses to evaluate stronger effects on incidence rates during COVID-19 surges.

Most studies published so far are of variable quality as they are limited to retrospective cohort studies. Moreover, they focus solely on COVID-19 patients, and lack standardized case definitions without differentiating between settings or specialties [28]. The current surveillance-based study has a retrospective design as well and hospitals performed the surveillance themselves, however by using standardized case definitions and large sample sizes from a fixed number of hospitals for several years, the results of our study may be more robust. Still, with our study design, we cannot fully explain (causal) reasons for the change in HAIs observed during the pandemic. Several hypotheses are possible, probably all contributing to some degree. In part, the increase in HAIs can be explained by the fact that hospitalisations were dominated by COVID-19 patients who may have been more vulnerable for HAIs and other co-infections due to immune dysregulation [29–32]. This is also reflected by the high antibiotic use observed in these patients, which will increase risk of antibiotic resistance. In Germany, there was no ICU overcrowding due to COVID-19 patients because of their high ICU bed capacity as compared with the Netherlands, and no increase in device-associated infections was observed in this country [33]. In addition, COVID-19 patients in general are more exposed to known risk factors for HAIs such as longer durations of mechanical ventilation, higher number of CVCs inserted, corticosteroid treatment, prone positioning, and longer lengths of stay [24]. Although not observed within this study, the composition of characteristics of remaining non-COVID-19 hospitalised patients is likely to be different than pre-pandemic, due to numerous elective procedures that were cancelled and postponed. Unfortunately, within the surveillance modules we only have limited patient- and clinical information, restricting the adjustment for casemix. Although we used data of a fixed set of hospitals and used the NNIS score and CVC applications to calculate the expected infection rates, we may not have completely addressed the shift in characteristics of the patient population during the pandemic. The increased CRBSI incidence in non-COVID-19 ICU patients may indicate that both a change in patient mix or the reorganization of care, such as changed IPC practices, modified use of personal protective equipment, and additional (unskilled ICU) temporary staff, may have contributed to the increased infection risk [5, 16, 34, 35]. To fully explain HAI dynamics in pandemic circumstances indicators describing the local healthcare context at institutional level are needed, such as patient characteristics, disruption of IPC practices, prescribing- and (microbiological) order practices, and antimicrobial resistance patterns [36].

## Conclusions

Summarized, we observed an increase in rates of CRBSI, VAP, gastro-intestinal- and central nervous system infections among hospitalised patients during the first pandemic year. Furthermore, CRBSI incidence was also increased in the non-COVID-19 ICU population during the pandemic. The full scope and driving factors of this change in HAIs need to be studied in more detail to be able to anticipate—from an infection prevention perspective—more adequately on future epidemics of COVID-19 or other severe acute respiratory infections.

## Supplementary Information

**Additional file 1:** Supplementary Figures and Tables.

## Data Availability

The datasets used and/or analysed during the current study are available, however, researchers should submit a data request to PREZIES (prezies@rivm.nl).

## Abbreviations

ASA: American Society of Anaesthesiologists
BSI: Bloodstream infection
CAUTI: Catheter-associated urinary tract infection
CRBSI: Catheter-related bloodstream infection
COVID-19: Coronavirus disease 2019
CVC: Central venous catheter
ICU: Intensive care unit
IPC: Infection prevention and control
IQR: Interquartile range
GI: Gastro-intestinal infection
HAI: Healthcare-associated infection
LRTI: Lower respiratory tract infections
NA: Not applicable
NNIS: National nosocomial infections surveillance system
PPS: Point prevalence survey
RTI: Respiratory tract infection
SSI: Surgical site infection
TPV: Total parenteral nutrition
UTI: Urinary tract infection
VAP: Ventilator-associated pneumonia
