# Supplementary material for: Healthcare-associated infections in Dutch hospitals during the COVID-19 pandemic
Source: Antimicrob Resist Infect Control. 2023 Jan 5;12:2. doi: 10.1186/s13756-022-01201-z (PMC9813899; doi:10.1186/s13756-022-01201-z)
Supplement: Supplementary file 1 — Additional file 1: Supplementary Figures and Tables. [file 13756_2022_1201_MOESM1_ESM.docx]

**SUPPLEMENTARY MATERIAL**

**FIGURES**

**Figure S1.** Calculations expected SSI rate (1a) and CRBSI per 1,000 catheter days (1b).

1a.

$$\boldsymbol{Expected infection percentage=}\frac{\boldsymbol{(}\left( \boldsymbol{A*Na} \right)\boldsymbol{+}\left( \boldsymbol{B*Nb} \right)\boldsymbol{+}\left( \boldsymbol{C*Nc} \right)\boldsymbol{+}\left( \boldsymbol{D*Nd} \right)\boldsymbol{+}\left( \boldsymbol{E*Ne} \right)\boldsymbol{)}}{\boldsymbol{(Na+Nb+Nc+Nd+Ne)}}$$

- A = National infection rate in 2016-feb2020 in NNIS category 0
- B = National infection rate in 2016-feb2020 in NNIS category 1
- C = National infection rate in 2016-feb2020 in NNIS category 2
- D = National infection rate in 2016-feb2020 in NNIS category 3
- E = National infection rate in 2016-feb2020 in NNIS category ‘unknown’
- Na = number of surgeries with NNIS category 0 in pandemic period
- Nb = number of surgeries with NNIS category 1 in pandemic period
- Nc = number of surgeries with NNIS category 2 in pandemic period
- Nd = number of surgeries with NNIS category 3 in pandemic period
- Ne = number of surgeries with NNIS category ‘unknown’ in pandemic period

1b.

$$\boldsymbol{Expected infection percentage=}\frac{\boldsymbol{(}\left( \boldsymbol{A*Na} \right)\boldsymbol{+}\left( \boldsymbol{B*Nb} \right)\boldsymbol{+}\left( \boldsymbol{C*Nc} \right)\boldsymbol{)}}{\boldsymbol{(Na+Nb+Nc)}}$$

- A = National infection rate in 2016-feb2020 in group of CVC for total parenteral nutrition (TPN) use
- B = National infection rate in 2016-feb2020 in group of CVC for dialysis use
- C = National infection rate in 2016-feb2020 of remaining categories (no TPN or dialysis)
- Na = number of CVCs for TPN use in pandemic period
- Nb = number of CVCs for dialysis in pandemic period
- Nc = number of CVCs of remaining categories (no TPN or dialysis) in pandemic period

**Figure S2.** Trends in total SSI rate after colorectal surgery and open versus closed procedures

**TABLES**

**Table S1.** Procedures under surveillance for surgical site infections

| **Type of surgery** | **Procedure** |
| --- | --- |
| Cardiothoracic surgery | Coronary artery bypass, Aortic valve surgery, ICD or pacemaker implantation |
| Mamma surgery | Mastectomy, Breast lumpectomy |
| Colon surgery | Colorectal resections, Cholecystectomy |
| Orthopaedic surgery | Arthroplasty of hip, Arthroplasty of knee |
| Obstetrics | Caesarean section |
| Neurosurgery | Laminectomy  Exploration or decompression of spinal cord through excision or incision into vertebral structures |

**Table S2.** Infection rates pre-pandemic, expected infection rates during pandemic, and observed infection rates during the pandemic for deep surgical site infections only

|  | **Pre-pandemic**  (% (95%-CI)) | **Predicted**  (% (95%-CI)) | **Pandemic**  (% (95%-CI)) |
| --- | --- | --- | --- |
| **Deep SSI incidence** |  |  |  |
| Overall | 1.0 (1.0 – 1.1) | 1.0 (0.9 – 1.1) | 0.9 (0.8 – 1.0) |
| Cardiothoracic surgery | 0.9 (0.7 – 1.2) | 0.9 (0.7 – 1.2) | 1.1 (0.6 – 1.9) |
| Mamma surgery | 0.4 (0.4 – 0.5) | 0.5 (0.3 – 0.8) | 0.9 (0.6 – 1.2) |
| Colon surgery | **2.7 (2.5 – 2.9)** | **2.9 (2.4 – 3.4)** | **1.8 (1.5 – 2.3)*** |
| Orthopaedic surgery | 0.9 (0.8 – 0.9) | 1.0 (0.8 – 1.1) | 0.8 (0.7 – 0.9) |
| Obstetrics | 0.2 (0.2 – 0.3) | 0.2 (0.1 – 0.5) | 0.2 (0.1 – 0.4) |
| Neurosurgery | 0.1 (0.0 – 0.3) | 0.1 (0.0 – 0.4) | 0.1 (0.0 – 0.8) |

**Table S3.** Distribution of HAI in COVID-19 patients versus non-COVID patients admitted to the hospital, October 2020 – December 2020.

|  | **COVID-19 patient** n=50 (n (%)) | **Non-COVID-19 patient** n=713 (n (%)) |
| --- | --- | --- |
| **HAIs (total)** | 6 | 3 |
| **SSIs** | 0 (0.0) | 1(33.3) |
| **VAPs** | 1 (16.7) | 1 (33.3) |
| **BSIs** | 4 (66.6) | 0 (0.0) |
| **UTIs** | 1 (16.7) | 1 (33.3) |

HAIs= healthcare-associated infections; SSIs= surgical site infections; VAP= ventilator associated pneumonia; BSIs= bloodstream infections; UTIs= urinary tract infections. Percentages are presented as proportion of total HAIs.
